# The evaluation of a health education campaign on the use of leave from work during pregnancy

**DOI:** 10.1186/1471-2458-10-694

**Published:** 2010-11-12

**Authors:** Giuseppe Mastrangelo, John H Lange, Emanuela Fadda, Ornella Agostini, Roberto Agnesi R, Andrea Bardin, Luca Cegolon

**Affiliations:** 1 Padua University, Department of Environmental Medicine and Public Health, Padua, Italy; 2 Envirosafe Training and Consultants, Pittsburgh, Pennsylvania, USA; 3 Provincial Directorate for Work, Venice, Italy; 4 Health and Safety at Work Service, Veneto PCT 16, Padua, Italy; 5 Imperial College London, School of Public Health, St. Mary's Campus, London, UK

## Abstract

**Background:**

The Italian Protective Maternity Legislation allows a woman to apply for early maternity leave from work during pregnancy if she is affected by health problems (option A) or if her working conditions are incompatible with pregnancy (option B). A community based health education program, implemented between 1995 to 1998 in North Eastern Italy, provided counseling (by a team of gynecologists, pediatricians, geneticists, psychologists and occupational physicians), and an information leaflet detailing the risks during pregnancy and the governmental benefits available to expectant mothers. This leaflet was distributed to women who were under occupational medical surveillance and to women attending any healthcare office and outpatient department and was also mailed to women working at home as shoemakers.

The effectiveness of this intervention has been evaluated in this investigation using an evidence based approach.

**Methods:**

A quasi-experimental design was adopted, applying several outcome measurements before (1989 to 1994) and after (1999 to 2005) the intervention. The outcome (ratio B/A) is the number of women receiving approval for B (circumstance where the pregnant woman is employed to undertake activities forbidden under the Article 7 of Law 151/2001, and it is impossible to change her duties) to those receiving approval for A (risky pregnancy due to personal medical conditions, Article 17 of the same Law). A linear regression coefficient (for B/A against years) was obtained separately for time periods "before" (1989-94) and "after" (1999-2005) the intervention program. The two regression coefficients were compared using a t-test.

**Results:**

The trend over-time for the ratio B/A was steady before the initial intervention (y = 0.008x - 16.087; t = 2.09; p > 0.05) then increased considerably (y = 0.0426x - 84.89; t = 19.55; p < 0.001) in coincidence with the start of the education campaign. There was a significant difference between the two regression coefficients (t = 7.58; p < 0.001).

**Conclusion:**

From a bureaucratic perspective Option B is far more complicated than A. In fact it implies an active approach involving an arrangement between the claimant and the employer, who has to certify to the relevant Authority that the woman's working conditions are incompatible with pregnancy. The increasing number of women availing of option B, as recommended, therefore suggests the suitability of such educational campaign(s).

## Background

In Italy, women have the right to paid leave from work for five months, two before and three after the delivery. By Italian law (151/2001), these five months can be extended to begin earlier in pregnancy (even immediately after pregnancy diagnosis) if the woman has recognized health problems or if her working conditions are incompatible with pregnancy. An employer must perform an assessment of the risks in the work environment for pregnant women. If the work environment is considered to be hazardous, the particular exposure in question should be reduced or work tasks changed. If neither is possible, women have a right to take an early leave from work during pregnancy. To obtain this benefit, women have to make an application to the Provincial Directorate for Work (DPL, Italian acronym) specifying whether there is:

• risks during pregnancy due to personal medical conditions (Letter a), Art. 17, Law 151/2001);

• a circumstance whereby the pregnant woman is employed to undertake activities forbidden under Article 7 of the same law, and it is impossible to change these duties (Letter b) and c), Art. 17, Law 151/2001).

The forbidden activities are those involving exposure to:

• chemicals: glues (in leather and shoe industry), paints, painting, glaze containing silica (ceramic, wood, metal working machines), metals (metal working machines and chemical industries), anesthetic gas (hospitals), solvents (industrial cleaning, printing, restoration);

• biological risk factors: contact with infectious material (laboratories and hospitals), contact with sick patients (hospitals), contact with children (nurseries, maternity wards);

• physical risk factors: lifting heavy objects, obligatory standing position for more than four hours per day, work on stairs, noise (textile industries), transport work, excessive fatigue or tension (daily or night shifts), exposure to ionizing radiations.

Domestic work is ignored: housewives do not qualify for this sort of employment protection.

Using the opportunities offered by the project "*Woman's Wellbeing*" launched by the Veneto Region (North Eastern Italy), a comprehensive health education campaign was implemented in the same Region from 1995 to 1998, consisting of:

• face-to-face counseling provided by a team of gynecologists, pediatricians, geneticists, psychologists and occupational physicians throughout the pregnancy - that included information on the adverse effects of environmental, occupational and behavioral exposures on the health of mother and fetus;

• an information leaflet illustrating health risks for pregnancy (as above) and the benefits assured by law distributed to workers during medical surveillance at workplaces, and to persons attending any surgery and outpatient department of the Primary Care Trust (PCT) 13 of the Veneto Region;

• another leaflet, informing on the health risks for pregnancy related to solvent containing products, was mailed to females working at home as shoemakers.

The intervention was carried out in an area (PCT 13, Veneto Region, North Eastern Italy), where numerous females of childbearing age were exposed to organic solvents in nearly 700 shoe factories. Some data collected in those years evidenced in many cases a failure to comply with certain hygiene requirements (for example, the absence of appropriate exhaust systems or incorrect mode of application of glues), and that there was little or no recourse to benefits granted by law 151/2001, in particular there were no or few cases of abstention from work during pregnancy when the problem was the unhealthy workplace (Letter b, Art. 17, Law 151/2001) [1].

In an earlier study, a before-and-after design was adopted to assess the intervention effectiveness [2]. The aim of this study is to reappraise the effectiveness of this community based health education program using a stronger design and an objective endpoint (the extended leave from work during pregnancy approved and granted by DPL).

## Methods

This study was carried out within the context of the project "*Woman's Wellbeing*", approved, funded and launched by the Veneto Region. Ethical approval was therefore not required.

A quasi-experimental design (time series design), which yields more sound information, was adopted by taking several outcome measurements before (baseline time trend) and after (second time trend) implementing the intervention program. To establish a trend, six measurements were performed before 1995 and six after 1998, as the heath education campaign was carried out during the time period 1995 to 1998.

The outcome in this study was leave from work during pregnancy. Relevant information was collected from the DPL records, local office of Venice. These records had name, address, and the type of action: Letter a), Letter b) or Letter c). These data were stored in a database where they were broken down by action taken: Letters a), b) or c), calendar year, and location of residence. The folder for year 2002 was not available, thus the observation time was extended until 2005. Letters b) and c) were pooled into a group named "B", with this quantity divided by group "A" (Letter a), and the B/A ratio plotted against calendar years. Using STATA 10, a median-band plot was obtained where the cross medians were graphed as a line plot. Furthermore, a linear regression coefficient (for B/A against years) was obtained separately for the period "before" (1989-94) and "after" (1999-2005).

The two regression coefficients (b_1989-94 _and b_1999-2005_) were compared using a method previously described [3], where an interaction term (product of years per a dummy variable being 0 for the baseline period and 1 for the second period) was used to test the null hypothesis H_0_: b_1989-94 _= b_1999-2005_. A significant t value indicates that b_1989-94 _is different from b_1999-2005_.

## Results

Table 1 displays the annual number of leaves from work during pregnancy issued by DPL from 1989 to 2005.

**Table 1:** Frequency distribution of leaves from work during pregnancy granted by the Provincial Directorate for Work from 1989 to 2005.

Year	A	B	B/A
1989	175	14	0.08

1990	178	14	0.08

1991	230	13	0.06

1992	253	34	0.13

1993	236	24	0.10

1994	262	28	0.11

1995	310	32	0.10

1996	346	65	0.19

1997	345	77	0.22

1998	416	94	0.26

1999	444	116	0.26

2000	468	146	0.31

2001	455	182	0.40

2002			

2003	568	271	0.48

2004	585	287	0.49

2005	520	282	0.54

A = leaves issued for personal medical conditions

B = leaves issued for occupational reasons

**Figure 1:**
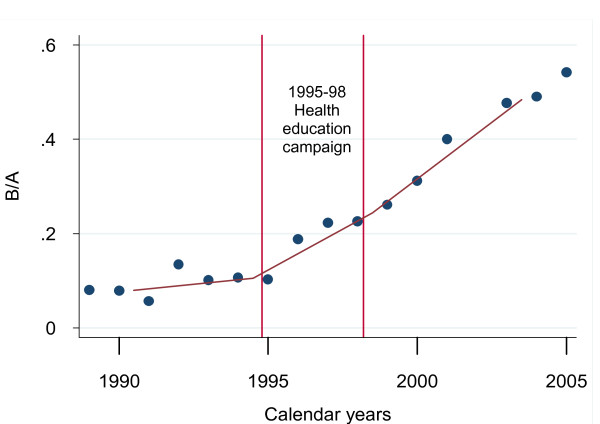
**Early maternity leaves allowed by the Provincial Directorate for Work (DPL): B/A^§ ^by years**. § “B” includes cases allowed by DPL under Letter b) and c) of Article 17, law 151/2001; “A” includes cases allowed by DPL under Letter a) of Art. 17, law 151/2001

Figure 1 shows that the ratio B/A was steady from 1989 up to 1995 (before the health education program was implemented), then increased considerably in line with the start of the health education campaign, constantly increasing even after the end of this program.

For B/A against years, the linear regression equation was 0.0081x - 16.087 (R^2^= 0.42; t = 2.09; df = 6; p > 0.05) for the period "before" (1989-94), and 0.0426x - 84.89 (R^2^= 0.98; t = 19.55; df = 8; p < 0.001) for the period "after" (1999-2005).

Table 2 shows that there was strong evidence (p < 0.001) of a difference between the two regression coefficients: b_1989-1994 _and b_1999-2005_.

**Table 2:** Comparison of two linear regression coefficients

TERMS	Regression coefficient (b)	Standard error (SE(b))	T test (b/SE(b))	p-value	95% Confidence Interval
**Calendar year**	0.008	0.004	2.3	0.042	0.0003 to 0.016
**Dummy variable**	-75.73	9.99	-7.58	0.001	-97.72 to -53.75
**Product**	0.038	0.005	7.58	0.001	0.027 to 0.049
**Constant**	-16.09	7.03	-2.29	0.043	-31.56 to -0.61

Dependent variable = ratio B/A♦. Predictor variables (TERMS): calendar years; a dummy variable (being 0 for the baseline period and 1 for the second period); the product of dummy with calendar years.

♦ "B" includes cases allowed by Provincial Work Directorate under Letter b) and c) of Article 17, Law 151/2001; "A" includes cases allowed by Provincial Work Directorate under Letter a) of Art. 17, Law 151/2001

## Discussion and Conclusions

In the years 1989-94, before the introduction of the above mentioned health educational project, there were no or few cases of abstention from work during pregnancy when the problem was an unhealthy workplace (Letter b), Art. 17, Law 151/2001). Women were normally opting for Letter a). This option is rather simple in nature, while the second option (Letters b) or c)) involves much more paperwork. In fact opting for "Letters b) or c)" involves more complex arrangements between the claimant and the employer, who has to certify to the relevant Authority that the woman's working conditions are incompatible with pregnancy. It is also necessary for women to provide more information and documents to enable the DPL to conclude that the claimant fulfils the conditions required by law. In the present study while option A saw some increase in use over the time period, there was a greater increase with option B. The increasing number of women availing of Letter b) of Art. 17, as recommended during the educational campaign, suggests the suitability of such educational intervention.

The Protective Maternity Legislation (PML) in Italy can be considered valuable [4], as it offers women various health benefits. A possible limitation of this Law is that it only provides protection after the diagnosis of pregnancy [5]. Spontaneous abortions or birth defects induced by occupational exposures during organogenesis or the peri-conception period (one month before pregnancy through the first trimester during pregnancy) are not prevented through this legislation [6]. This is therefore a limitation applying also to the effectiveness of this health education campaign.

It is possible to strengthen the before-and- after design further by combining two approaches: taking more measurements and adding a non-randomized control group [7]. This multiple time series design could reduce interference by external circumstances (for example, the increasing general awareness of reproductive hazards at the workplace), because they often apply to both the control group and the intervention group. It therefore allows a separation of the effect of the intervention from that of other circumstances. Specifically in this study the fact that the ratio continues to rise after the start of the campaign, could also signify that the DPL began approving a greater percentage of "B" applications at about the same time as the heath education campaign was conducted, perhaps as a result of the DPL reviewers becoming more sympathetic to these "B" requests. This history threat has to be taken into account given the fact that the number of applications (the denominator) is unknown.

The lack of a control group prevents us being in a position to attribute the findings of the present study to the health awareness campaign alone.

Another limitation is that only regularly employed women generally benefit from PML. Despite a lack of relevant data, it could be presumed that there are inequalities among eligible workers: women with less qualified jobs and those employed in the private sector were less likely to benefit from these protective measures [8]. Furthermore PML covers some but not all pregnant workers, as it ignores domestic workers and the tendency to remove the pregnant workers rather than to modify their working conditions or lower the workplace exposure [9,10].

## Competing interests

The authors declare that they have no competing interests.

## Ethical Approval

Not required. This study is an assessment of efficacy of a health education program approved and funded by the Veneto Region, called "*Woman's Wellbeing*".

## Authors' contributions

GM and RA conceived the idea and developed the paper, LC contributing to the drafting of the paper and the statistical analysis, JHL and EF contributing to the drafting of the paper, OA and AB collected the data and contributed to the literature search. All authors read and approved the final manuscript.

## Pre-publication history

The pre-publication history for this paper can be accessed here:

http://www.biomedcentral.com/1471-2458/10/694/prepub
